# Prevalence of *Toxoplasma gondii *infection in *Myocastor coypus *in a protected Italian wetland

**DOI:** 10.1186/1756-3305-4-240

**Published:** 2011-12-23

**Authors:** Simona Nardoni, Maria C Angelici, Linda Mugnaini, Francesca Mancianti

**Affiliations:** 1 Dipartimento di Patologia Animale, Profilassi ed Igiene degli Alimenti, viale delle Piagge 2, 56100 Pisa, Italy; 2 Dipartimento di Ambiente e Connessa Prevenzione Primaria, Istituto Superiore di Sanità, Roma, Italy

**Keywords:** *Toxoplasma gondii*, *Myocastor coypus*, wetland, Italy, seroprevalence, n-PCR.

## Abstract

**Background:**

*Toxoplasma gondii *is the causative agent for a major zoonosis with cosmopolitan distribution. Water has been implicated in outbreaks of toxoplasmosis in recent years. Coypus (*Myocastor coypus*), commonly nutria, are large semi-aquatic invasive rodents, naturalized throughout European countries, including most wetlands of Central Italy. The habitat of these animals is both terrestrial and aquatic, making them a species highly exposed to the parasite.

**Findings:**

The occurrence of the infection was evaluated using a modified agglutination test (MAT) in 74 adult coypus from a naturalized population living in a wetland of Central Italy. Nested PCR (n-PCR) assay was carried out on some of them. Positive *T. gondii *MAT results were found in 44 animals (59·4%), 30 males (68·2%) and 14 females (31·8%). Antibody titers were ranging from 20 to 40960, while 12 out of 23 (52·2%), examined animals, 8 males (66·7%) and 4 females (33·3%), resulted positive to n-PCR. All n-PCR positive animals were seropositive, showing antibody titers ranging from 640 to 40960.

**Conclusions:**

Our results indicate that examined animals are heavily parasitized with *Toxoplasma*. This suggests that coypus could be a reservoir of this parasite, because they can be eaten both by scavenger animals and by humans, and that these animals would play a role in maintaining the cycle of *T. gondii*.

## Findings

*Toxoplasma gondii *is an obligatory intracellular protozoan parasite which can infect all warm-blooded animals and is the aetiological agent of toxoplasmosis, a major zoonosis. *T. gondii *has adapted to an oocyst-oral cycle in herbivores [1,2]. Felines are known to act as definitive hosts and they can shed millions of oocysts in the environment. After a period they sporulate and become highly infectious and resistant to environmental influences, being able to maintain their infectivity in moist soil as well as on fruits and vegetables for long periods [3,4]. Mechanical spread of oocysts by flies, cockroaches, dung beetles and earthworms represents an additional route of infection [1]. The role of filter feeder mussels as casual reservoir has also been established [5,6].

Coypus (*Myocastor coypus*), commonly nutria, are large semi-aquatic invasive rodents, naturalized throughout European countries, including most wetlands of Italy. Coypu is ecologically associated with people due to changes in the ecosystem caused by human activities. It adapts to a wide variety of environmental conditions and persists in areas previously claimed to be unsuitable. Nutria habitat is the semi aquatic environment occurring at the boundary between land and permanent water. These rodents require water and abundant emergent aquatic vegetation for feeding, are almost entirely herbivorous and eat animals (mostly insects) incidentally, when they feed on plants. Freshwater mussels and crustaceans are also occasionally eaten. Nutria are regarded as a pest, and they can be infected with several pathogens and parasites transmissible to humans, livestock, and pets [7].

Toxoplasmosis is a common infection in both farm-raised and free-ranging nutria [8]. Animals challenged with *Toxoplasma *pathogenic strains do not develop referable clinical signs, so this species can be considered highly resistant to the parasitosis [9]. Contextually, coypus exhibit a detectable and specific antibody response, and could be putative candidates to monitor the burden of *T. gondii *oocysts in the environment. The habitat of these animals is both terrestrial and aquatic, making coypu a species highly exposed to the parasite. It is well stated that *T. gondii *can be spread by food and soil. Water has been implicated in outbreaks of toxoplasmosis in recent years [10]. The possibility that ingestion of untreated water could be a risk factor has been evaluated in the epidemiology of this infection, considering that a large proportion of human infections could not be explained by contaminated food or soil exposure [11]. The objective of the present study was to evaluate the prevalence of *T. gondii *in a naturalized population of nutria living in moist areas of Central Italy, by modified agglutination test (MAT) and n-PCR assays.

Study area - All coypus captures were located within Fucecchio Marshes, a protected wetland area (longitude 10° 48' 10" east; latitude 43° 44' 0" north) in Northern Tuscany, comprising part of the provinces of Pistoia and Florence. It is the largest inland wetland in Italy, extending for about 1.800 ha. About 230 ha inside this area constitutes a natural reserve. The Centre for Research, Documentation and Promotion of Fucecchio Marshes promotes the conservation and improvement of the area, managing a Laboratory for Environmental Education. Guided tours for schools and adult groups, permanent education and refresher courses for teachers are organized, so allowing the fruition of the Nature Reserve both with educative and touristic purposes.

Animals sampled - A total of 74 mature coypus aged more than 8 months, of both genders (48 males and 26 females) were trapped, then euthanized by intracardiac puncture of Tanax^® ^(embutramide, mebenzonium iodide, and tetracaine hydrochloride solution), according to Italian legislation during a screening program to monitor the occurrence of *Leptospira *sp. infection in wildlife. The animals appeared to be in good health status, with no signs referable to toxoplasmosis. Blood samples were collected immediately after killing as described by Bollo et al. [7] for serologic examination. When possible, portions of kidney were collected for molecular purposes. Serum samples were screened for *T. gondii*-specific IgG with a modified agglutination test (MAT), performed by a direct agglutination commercial kit (Toxo-Screen DA^®^, bioMérieux, Rome, Italy). Sera were tested with formalin-fixed whole tachyzoites as antigen. Two-fold dilutions were achieved and the cut-off titer was 1/20 as recommended by Dubey et al. [12].

Nested-PCR (n-PCR) assay - Twenty-three kidney samples were used to detect parasite DNA, using a nested-PCR protocol. DNA purification from samples was performed on a 200 mg sample using a DNA extraction kit (DNAeasy Blood and Tissue Kit, Qiagen, Germany), according to the manufacturer's instructions. After extraction, DNA was stored at -20°C until use. Two pairs of oligonucleotide primers directed against the B1 gene of *T. gondii *were used to perform a nested PCR using purified *T. gondii *DNA as a template [13]. In the first-round amplification, PCR mixture contained 10 mM Tris-HCl, pH 8.3 (at 25°C), 50 mM KCl, 2 mM MgCl2, 0.1 μM of each outer primer (GGAACTGCATCCGTTCATGAG and TCTTTAAAGCGTTCGTGGTC), 0.1 mM each dNTP, 1.25 U *Taq *DNA polymerase (AmpliTaq^® ^Gold, Roche), in a final volume of 20 μl. Reactions were cycled in a thermocycler (MyCycler, Bio-Rad, Hercules, California) 40 times, with denaturation at 93°C for 10 seconds followed by annealing at 57°C for 10 seconds and finally an extension step at 72°C for 30 seconds. Nested reaction contained 1 μl of first-round PCR product added as a template, 10 mM Tris-HCl, pH 8.3 (at 25°C), 50 mM KCl, 3 mM MgCl2, 0.5 μM each inner primer (TGCATAGGTTGCAGTCACTG and GGCGACCAATCTGCGAATACACC), 0.1 mM each dNTP, and 1 U *Taq *DNA polymerase in a final volume of 20 μl. N-PCR was cycled 40 times using a denaturation step of 93°C for 10 seconds, followed by annealing at 62.5°C for 10 seconds and extension at 72°C for 15 seconds. Negative control samples from first-round amplification and an additional second-round negative control of sterile water were included in the nested reactions. Contamination by amplicons was avoided by using separate rooms and material as well as decontamination procedures (UV exposure and bleaching of materials and surfaces). Cross-contamination was monitored by negative controls for sample extraction and PCR solutions. Amplification reactions were analyzed by 1.5% agarose gel electrophoresis, and visualized under UV light. Samples were scored as positive when a n-PCR product of 96 bp was detected.

Data analysis - Chi-square test was used to evaluate parasitological results relative to gender. A probability *(P) *value < 0.01 was considered as statistically significant.

Positive *T. gondii *MAT results were found in 44 animals (59·4%), 30 males (68·2%) and 14 females (31·8%). Antibody titers ranged from 20 to 40960, while 12 of 23 animals, 8 males (66·7%) and 4 females (33·3%) resulted n-PCR positive. All n-PCR positive animals were seropositive, showing antibody titers ranging from 640 to 40960. There was no statistically significant difference for the seroprevalence rates between males and females. MAT antibody titers as compared to PCR results in examined coypus are presented in Table 1. In the present study more than 50% of the examined coypus showed high antibody titres against *T. gondii*. Data available from the literature referring to free ranging coypus reported lower seroprevalence values, such as 7% in the USA [8] and 36·6% in Italy [7]. This difference could be due to the diversity of environmental conditions of study areas. The survey of Bollo et al. [7] was carried out in a natural protected area characterized by larger rivers with high flow and fast moving water, which could sweep away parasitic forms. In our study, the abundance of confined stagnant waters and the high degree of anthropization with a conspicuous presence of cats, could lead to oocysts spreading in the environment. The high seroprevalence of anti-*T. gondii *antibodies in coypus studied indicate that this parasite is present in their habitat, and the occurrence of parasites in coypu tissues could probably reflect this distribution. Serologic findings were corroborated by molecular investigations, considering that 12 of 23 kidneys (52·2%) scored positive for *T. gondii *DNA. Unfortunately, in this study the commonest sites of cyst formation i.e. heart and brain [14,15], were not available as first choice tissues for detection of toxoplasmic DNA. The high positive rate for the PCR test obtained in the present work could suggest that the examined nutria population was heavily parasitized. On the other hand, the agreement between direct diagnosis of parasite in the kidney samples and the highest serum titers would be indicative of parasitaemia status and of active infection in the examined animals. Similar surveys carried out on foxes reported that molecular techniques were less sensitive than serology [16].

**Table 1:** MAT antibody titers as compared to PCR results in examined coypus.

MAT titer	20	40	80	160	320	640	2560	5120	10240	20480	40960	Total
MAT positive animals	4	6	6	3	3	1	2	3	6	6	4	44

PCR positive animals						1	1	2	3	3	2	12

PCR negative animals		1		1				1	1			4

Executed PCR assays	0	1	0	1	0	1	1	3	4	3	2	16

Our results seem to confirm that the parasitosis is widespread in these animals, which may lead us to assume that infection with oocysts is acquired from feeding on mollusks and from drinking infected water so becoming a potential reservoir of the parasite. Coypus are, in fact, a possible food resource for scavenger animals, but also for humans, considering that meat from nutria is commonly consumed in South America and in some European countries [17]. In addition, the coypu infection by *Toxoplasma *would indicate the local occurrence of the parasitic infective cycle, also in consideration that Fucecchio Marshes Natural Reserve is visited by school children for a variety of nature activities.

The results of the present survey indicate that *Toxoplasma *infection is widespread in coypus of Fucecchio Marshes Natural Reserve. Considering that all the animals included in the study appeared in good health status, and on the basis of literature [9], infected coypus show no apparent signs of illness, and could act as a reservoir for infection.

## Competing interests

The authors declare that they have no competing interests.

## Authors' contributions

FM designed, managed and conducted the study. FM and SN analyzed, interpreted data and drafted the manuscript. MCA carried out PCR detection and revised the results of the manuscript. SN and LM performed serological analysis. All authors read and approved the final manuscript.
